# The SWC4 subunit of the SWR1 chromatin remodeling complex is involved in varying virulence of *Metarhizium brunneum* isolates offering role of epigenetic regulation of pathogenicity

**DOI:** 10.1080/21505594.2022.2101210

**Published:** 2022-07-26

**Authors:** Victoria Reingold, Alessia Staropoli, Adi Faigenboim, Marcel Maymone, Sabina Matveev, Ravindran Keppanan, Murad Ghanim, Francesco Vinale, Dana Ment

**Affiliations:** a Department of Plant Pathology and Weed Research, Agricultural Research Organization (ARO), The Volcani Institute, Rishon LeZion, Israel; b The Robert H. Smith Faculty of Agriculture, Food & Environment, The Hebrew University of Jerusalem, Rehovot, Israel; c Department of Agricultural Sciences, University of Naples Federico II, Portici, Italy; d Institute for Sustainable Plant Protection, National Research Council, Portici, Italy; e Institute of Plant Science, ARO, The Volcani Institute, Rishon Le Zion, Israel; f Department of Entomology, Nematology and Chemistry Units, ARO, The Volcani Institute, Rishon LeZion, Israel; g Department of Veterinary Medicine and Animal Production, University of Naples Federico II, Naples, Italy

**Keywords:** Entomopathogenic fungi, *Galleria mellonella*, adenine methylation, secondary metabolites, Cas9-Mediated mutagenesis, pathogenicity

## Abstract

The host – pathogen interaction is a multifactorial process subject to a co-evolutionary arms race consisting of rapid changes in both host and pathogen, controlled at the genetic and epigenetic levels. Previously, we showed intra-species variation in disease progression and pathogenicity in aphids for *Metarhizium brunneum* isolates MbK and Mb7. Herein, we compared genomic, epigenetic, and metabolomic variations between these isolates and their effects on pathogenicity. Genomic variation could not completely explain the observed differences between the isolates. However, differential N6-adenine methylation (6 mA) and its correlation to reduced expression of the essential SWC4 subunit of SWR1 chromatin-remodelling complex (SWR1-C) led us to hypothesize a role for *swc4* in the varying pathogenicity. Mutagenesis of the essential *swc4* gene in MbKisolate resulted in reduction of secondary-metabolite (SM) secretion and impaired virulence in *Galleria mellonella*. Our results suggest the role of SWC4 in the regulation of SMs and the role of both SWC4 and SWR1-C in virulence of *M. brunneum* isolates. A better understanding of epigenetic regulation of SM production and secretion in entomopathogenic fungi may enable theirmanipulation for better biocontrol performance, and expand possibilities for environmentally friendly pest control.

## Introduction

The host–pathogen interaction is a multifactorial process subject to a co-evolutionary arms race [1]. This arms race consists of rapid changes in not only the genomes, but also the behaviour of both the host and the pathogen, as each responds to their partner’s attacks [2]. Entomopathogenic fungi (EPF) are an environmentally friendly alternative to synthetic pesticides, and major players in integrated pest management towards reducing the use of chemical insecticides [3]. *Metarhizium* spp. (Hypocreales: Clavicipitaceae) are among the most prevalent and best-studied EPF. Moreover, *Metarhizium* spp. are a model system for host–pathogen interactions and a resource for biotechnological tools [4–6].

The life cycle of hypocrealean fungi, such as *Metarhizium*, can be divided into two major stages: the pathogenic lifestyle stage, involving pathogenic interactions of the asexual conidia with invertebrate hosts, and the saprophytic lifestyle stage, involving proliferation in the rhizosphere and on soil organic matter as a saprophyte. On susceptible hosts, the pathogenic lifestyle of hypocrealean EPF consists of primary infection on the host cuticle (adhesion, germination), followed by later stages of infection (penetration, colonization, conidiogenesis) [7]. Infection progression requires the pathogen to be adapted to, and compatible with the host, and to possess the genes, proteins and secondary metabolites (SMs) needed for successful infection [8]. Species of *Metarhizium* secrete SMs which assist the fungi to evade and weaken insect’s immune system, such as destruxins [9].

Variation in host susceptibility can be attributed to both fungal and host characteristics [1,10,11]. In fungi, the penetration process has been extensively studied and shown to include the production of a versatile set of cuticle degrading enzymes [12]. In many cases, the host is unable to resist mycosis once penetration of the cuticle occurs [13–15].

Fungal infection of insect hosts progresses rapidly: studies have found that within 24 h of inoculation, there are changes in host gene expression in response to germination on the outer cuticle [16]. The regulation of pathogen attack and host counterattack is largely uncharacterized. Whereas spontaneous mutations in virulence and resistance genes are a key driver of this co-evolutionary arms race, epigenetic changes provide a mechanism for more rapid, stable shifts in gene expression, allowing for faster adaptation to evolutionary innovation in the partner.

Studies have shown the importance of epigenetic regulation, particularly in asexual organisms [17,18]. Epigenetic marks such as DNA methylation and histone acetylation can provide infected hosts with resistance to a fungal pathogen that can be transferred to subsequent generations [19]. Studies in recent years have demonstrated the importance of histone modifications in host – pathogen interactions [6,20]. Furthermore, the epigenetic regulators themselves, such as methyltransferases, can be strongly manipulated during infection and in turn, affect a cascade of induction and suppression of genes involved in virulence and pathogenicity [21].

The most common epigenetic DNA modification in eukaryotes is 5-methylcytosine (m5C), which has been studied in a variety of models and conditions. This modification is known to occur across the genomes of organisms from several kingdoms and control various processes [22]. N6-adenine methylation (6 mA), a well-known DNA modification in bacterial genomes, was recently identified at various levels in eukaryotic genomes [23,24], and shown to occur mainly in expressed genes [23]. In bacteria, 6 mA plays an essential role in virulence and protection from foreign DNA [25]. However, its role and mode of action in eukaryotes have yet to be determined.

Variations in EPF species’ virulence to different hosts can be attributed to the fungi’s “toolkit” and the level of compatibility with the host, and later, to their ability to evade the host’s immune system. For successful infection, fungi secrete SMs, which are organic compounds comprising antimicrobial and immunosuppressant compounds [26]. These can cause mortality even when used as purified extracts [27] and are one of the main factors controlling fungal compatibility with certain hosts [28,29]. Many SM gene clusters have been putatively found in EPF genomes, but their regulation mechanisms and final products are unknown [29–31]. The role of chromatin-modification and remodelling complexes in controlling SM production and fungal virulence has been elucidated [32–34]. One of the most interesting chromatin-remodelling complexes, which has yet to be demonstrated as a SM regulator in fungi, is the SWR1 complex (SWR1-C), which is involved in the deposition of histone variant H2A.Z into the nucleosome [32]. Its wide presence in eukaryotes, from humans (as SRCAP) to yeast, demonstrates its essential nature, as it plays critical roles in DNA repair and transcription regulation [32,35,36].

The proper selection of fungal strain is crucial for the effectiveness of integrated pest management against the desired insect pest [28,37]. Different EPF species have varying degrees of virulence depending on the host being challenged, especially when specialist strains are used. However, generalist fungal strains may also perform differently on different hosts [13,14,38]. Recently, we revealed that not only inter-species, but also intra-specie variations in *M. brunneum* affect virulence ability [7]. In the present study, we further explored the basis for better performance of one isolate over the other in the two *M. brunneum* isolates MbK and Mb7. Here, we focused on the role of epigenetic regulation and demonstrate how epigenetic regulators affect pathogenicity in *M. brunneum*.

## Materials and methods

### Fungal growth and maintenance

Two selected isolates of *M. brunneum*, Mb7 and MbK, were used in this study [7]. Fungi were cultured on SDA (Difco) plates at 28 ± 0.5°C in an incubator. Sporulation occurred mainly during the first 14 d of incubation.

### Genomic DNA extraction from fungal material

Conidia were collected from SDA plates using a sterile loop and placed in 2-mL screw-cap tubes (for the extraction of fungal gDNA from infected *Myzus persicae* aphids: 7 DPI, aphids [7] were collected in a 2-mL screw-cap tube and a similar extraction was carried out). Samples were ground with metal beads in the presence of lysis buffer (Lucigen, USA) using a Geno/Grinder (SPEX SamplePrep, USA) at 6500 oscillations/min for 2 min. Then, the samples were subjected to gDNA extraction using MasterPure Yeast DNA Extraction Kit (Lucigen) according to the manufacturer’s instructions. Samples were analysed by Nanodrop (Thermo Fisher Scientific, USA) and separated on a 1% agarose gel in the presence of ethidium bromide. Samples were maintained at −20°C until used.

### Next-generation sequencing and bioinformatics analysis

Genomic DNA samples extracted from Mb7 and MbK fungal isolates were further purified using gDNA clean & concentrator Kit (Zymo, USA) and served for library construction and Illumina 150- paired end next-generation sequencing (Sequencing core, University of Illinois, USA). Raw paired-reads were subjected to a filtering and cleaning procedure. The FASTX Toolkit (http://hannonlab.cshl.edu/fastx_toolkit/index.html, version 0.0.13.2) was used to trim read-end nucleotides with quality scores <30, using the fastq_quality_trimmer, and to remove reads with less than 70% base pairs with a quality score ≤30 using the Fastq_quality_filter. Clean reads (20.5 and 14.9 paired-end million reads for Mb7 and MbK, respectively) were mapped to the *Metarhizium brunneum* (GCA_013426205.1_ASM1342620v1) genome using the Burrows – Wheeler Aligner (BWA) software 0.7.12-r1039 [39]. The resulting mapping files were processed using SAMtools/Picard tool [http://broadinstitute.github.io/picard/, version 1.78 [40]] for adding read group information, sorting, marking duplicates and indexing. Then, the local realignment process for locally realigning reads was performed so that the number of mismatching bases is minimized across all the reads using the RealignerTargetCreator and IndelRealigner of the Genome Analysis Toolkit version v4.1.9.0 [GATK; http://www.broadinstitute.org/gatk/ [41]]. Finally, the variant calling procedure was performed using HaplotypeCaller of the GATK toolkit (https://gatk.broadinstitute.org/hc/en-us) developed by Broad Institute of MIT and Harvard (Cambridge, MA, USA). SnpEff tool [version 5.0d [42]] was used for annotations and predictions the effects of genetic variants based on the annotation downloaded from the NCBI database (https://ftp.ncbi.nlm.nih.gov/genomes/all/GCA/013/426/205/GCA_013426205.1_ASM1342620v1/GCA_013426205.1_ASM1342620v1_genomic.gff.gz). Clean paired-end reads were assembled de novo using SPAdes toolkit [v3.14.1 [43]]. Scaffold above 2000 bp were analysed for gene prediction using OmicsBox (v2.0.36) based on AUGUSTUS software [44]. The predicated proteins were used as a query term for a search of the NCBI non-redundant (nr) protein database that was carried out with the DIAMOND program [45]. The search results were imported into Blast2GO version 4.0 [46] for gene ontology (GO) assignments. OrthoFinder program (v2.3.3) was used to identify orthologous groups of proteins among the two *de-novo* assemblies (MbK and Mb7) and the reference genome (GCA_013426205.1) [47].

### Data availability

The raw paired-end reads were deposited in the National Center for Biotechnology Information BioProject database (http://www.ncbi.nlm.nih.gov/bioproject) with IDs PRJNA819239 and PRJNA819240 for MbK and Mb7, respectively.

### Methylated-adenine library construction

Three gDNA extractions from harvested conidia were performed to construct the methylated-adenine libraries. The conidia were collected from (1)14-d-old SDA plates with MbK, (2) 14-d-old SDA plates with Mb7, both serving as saprophytic growth samples, and (3) pathogenic sample gDNA from 10 individual *Myzus persicae* aphids. Libraries were constructed as described previously [48] with some modifications. Briefly, extracted gDNA was predigested with *EcoRI* (Thermo Fisher Scientific) and subjected to further digestion with *DpnI* (Thermo Fisher Scientific). Digested gDNA served as the template for library construction using blunt-end adaptors (S1 Table, AdRt and AdRb) and for PCR amplification using the adapter’s complement primers (S1 Table, AdR_PCR), then was separated on and extracted from a 1% agarose gel using the ZymoClean Gel DNA Recovery Kit (Zymo). Ligation was carried out using the CloneJET PCR Cloning Kit (Thermo Fisher Scientific), followed by transformation into DH5a competent cells (Bio-Lab, Israel). Colony PCR was conducted using the primers provided with CloneJET and positive colonies (containing an insert) were submitted to Sanger sequencing (hylabs, Israel).

### Validation of *swc4* gene adenine methylation

Conidial gDNA was extracted from Mb7 and Mbk isolates harvested from 14-d-old SDA cultures, and evaluated by NanoDrop spectrophotometer and 1% agarose gel separation. The gDNA was diluted to 500 ng with ultrapure water (Bio-Lab) and then digested with *DpnI*. Untreated gDNA served as a control. The digested material served for PCR amplification using specific primers encompassing a region within the *swc4* gene (S1 Table, SWC4_1 + 2 or SWC4_3 + 4). Amplicons were analysed by separation on a 1% agarose gel in the presence of ethidium bromide.

### RNA extraction from fungal material and cDNA synthesis

Conidia were harvested from SDA plates in the presence of 0.01% (v/v) Triton X-100 in distilled water. Samples were flash-frozen in liquid nitrogen with the addition of TRIzol (Thermo Fisher Scientific) and ground with metal beads in the Geno/Grinder at 6,500 oscillations/min for 3 min. Then, total RNA extraction was carried out according to the TRIzol manufacturer’s protocol. The extracted samples were treated with DNaseI (Thermo Fisher Scientific) to remove gDNA according to the manufacturer’s instructions. Total RNA was analysed by NanoDrop spectrophotometer and 500 ng were taken for cDNA synthesis with an oligo dT primer using the Verso cDNA Synthesis Kit (Thermo Fisher Scientific) according to the manufacturer’s instructions.

### Differential expression determination by RT-qPCR

The obtained cDNA was subjected to qRT-PCR using Fast SYBR Green Master Mix (Thermo Fisher Scientific) and *tef* as the reference gene using specific primers [S1 Table, qMb_TEF F and R [49]]. The primers for amplifying *swc4* (S1 Table, qMb_SWC4 F and R; qMb_SWC4 p.c. F and R) and *swr1* (S1 Table, qMb_SWR1 F and R; qMb_SWR1 p.c. F and R) were examined for their efficiency by conducting standard curve analysis and then served for qRT-PCR analysis for differential expression. The qRT-PCR analyses were conducted three times for each comparison. The relative expression levels were calculated using the 2^−ddCt^ method [50]. The relative expression results were statistically analysed by standard least squares analysis by restricted maximum likelihood (REML), using repeat as random effect, followed by post hoc comparison by Student’s *t*-test using JMP Pro software version 16.0.0 (SAS Institute Inc.).

### CRISPR single guide (sg) RNA design for *swc4* and *swr1* gene mutagenesis, Cas9 reaction setup

The CRISPR mutagenesis protocol was based on Davis et al. [51]. Single guide RNA targeting genes of interest was designed using Benchling [Biology Software (2021) Retrieved from https://benchling.com] based on Cas9 cutting efficiency [52]. The sgRNA template was synthesized as a primer (S1 Table, SWC4_sg132 or SWR1_sg1979) and served for sgRNA synthesis using the EnGen sgRNA Synthesis Kit (NEB, USA) following the manufacturer’s instructions. The sgRNA was then purified using the RNA Clean & Concentrator-25 Kit (Zymo) following the manufacturer’s instructions. RNA concentration and quality were assessed by NanoDrop spectrophotometer. For each CRISPR reaction: 2.5 μL concentrated sgRNA, 2 μL of EnGen Spy Cas9 NLS (NEB) in the presence of buffer 3.1 (NEB) in a final volume of 5 μL were incubated at 37°C for 30 min and kept on ice until use.

### Selective double-stranded (ds) DNA marker production

Using pSK1019 (kindly provided by Prof. Seogchan Kang at The Pennsylvania State University, USA) as a template, the regions of the *egfp* reporter gene (including the promoter from the *Cochliobolus heterostrophus* glyceraldehyde 3-phosphate dehydrogenase gene) and *hph* (including the promoter from *Aspergillus nidulans trpC*) were amplified using primers pSK_DS_HYG_F and pSK_DS_eGFP_R (S1 Table), designed to overlap with ca. 100 bp upstream and downstream of the region resulting in a 2946-bp amplicon. For the PCR: 1 μL of pSK1019 plasmid (20 ng/μL) and 1 μL of each of the primers (10 μM) were amplified using Phusion High-Fidelity DNA Polymerase (NEB) in a final volume of 20 μL according to the manufacturer’s instructions, under the following conditions: 98°C for 30 s followed by 35 cycles of: 98°C for 10 s−60°C for 10 s−72°C for 3 min, and a final extension step at 72°C for 5 min. The PCR product was separated on a 1% agarose gel, and purified using the Zymoclean Gel DNA Recovery Kit. The concentrated product (ca. 200 ng/μL) served as the selective dsDNA marker for CRISPR mutagenesis.

### Fungal protoplast transformation and colony selection

Protoplasts of *M. brunneum* MbK were prepared as described previously [51,53] with some modifications. Briefly, conidia harvested from a 7-d-old culture of isolate MbK served as a fungal starter, which was grown for 3 d in malt extract broth media. Then, the mycelium was filtered through miracloth (Merck Millipore, USA), washed with sterile distilled water and with KCl/CaCl_2_ buffer (KCl 89.92 g/L, CaCl_2_ 7.35 g/L), and transferred into a flask containing 20 mL of enzyme solution [50 mL KCl/CaCl_2_ buffer containing 200 mg lysing enzyme (Sigma, USA), 150 mg driselase (Sigma), 15 mg lyticase (Sigma), 10 mg bovine serum albumin (Sigma), and 10 mg yatalase (Takara Bio, Japan)] and incubated for 2–3 h at 28°C with shaking at 90 rpm/min. Protoplasts were separated from the mycelial debris by filtration through miracloth and washed twice with KCl/CaCl_2_ buffer, then suspended in 750 μL STC buffer solution (1.2 M sorbitol, 100 mM Tris pH 7.5, 0.05 M CaCl_2_·2 H_2_O), and the concentration was estimated by haemocytometer. STC buffer was added to obtain a final concentration of 5 × 10^7^ protoplasts/mL. A 1/4 volume of PEG solution [two volumes of 37% v/v PEG 8000, with one volume of 3X PEG amendments (1.8 M KCl, 150 mM, CaCl_2_, 150 mM Tris pH 7.4)] was added to the protoplasts in the STC buffer. For transformation, 125 μL of protoplast solution was incubated with 5 μL of a sgRNA – Cas9 mix and 5 μL of the selective dsDNA on ice for 30 min. Then, 1 mL of PEG solution was added and the mixture was incubated for an additional 20 min at room temperature. Transformed protoplasts were plated on 10 mL growth medium (sucrose 239.4 g/L, yeast extract 0.5 g/L) and left to recover for 2–3 h, then the top layer of 20 mL media containing Hygromycin B Gold antibiotic (InvivoGen, France) at a final concentration of 500 μg/mL per plate, was added and left to dry. Plates were incubated at 28°C for 2 weeks, during which time emerged colonies were collected and transferred to SDA plates containing 200 μg/mL Hygromycin B Gold. Colonies were then single-spore isolated and served for gDNA extraction followed by PCR amplifications and sequencing of the *swc4* or *swr1* genes.

### Morphological characterization of mutants

Fungal isolates Mb7 and MbK and the *swc4* and *swr1* mutants generated from MbK were used for morphological analysis of growth. Using a 200-µL sterile tip, a round piece of fully sporulated SDA (5 mm) was collected from 14-d-old SDA plates, placed in 5 mL 0.01% Triton X-100 and vigorously vortexed to obtain a conidial suspension. The conidial concentration was estimated by haemocytometer. Each suspension was then diluted to a final concentration of 1 × 10^6^ conidia/mL. A 30-µL aliquot of the diluted suspension was spread on an SDA plate for germination assay, which was examined 16 h postplating. Simultaneously, 30-µL aliquots were placed in the middle of another three SDA plates, without spreading, for radial growth assay. Plates were maintained as detailed in the “Fungal growth and maintenance” section and growth was documented at 7 and 14 d postplating. The assays were performed at least three independent times for each fungal isolate and mutant. Statistical analysis was performed using JMP Pro software version 16.0.0. Conidial concentration, germination and radial growth were subjected to standard least squares analysis by restricted maximum likelihood (REML) method followed by Dunnett’s test for conidial concentration and germination using MbK as control group for comparison. Tukey’s HSD test for multiple comparisons among means was used for radial growth.

## Metabolomic analysis

### Fungal growth conditions

Fourteen-day-old fungal SDA plates of Mb7, MbK, and MbK-*swc4*-mutant served as the source material for the metabolomics analysis and metabolite assay. A 1 cm × 1 cm piece from each plate was placed in a 250-mL flask containing 100 mL of sterile SDB (Difco). Inoculated flasks were sealed with sterile aluminium foil and cultured at 28 ± 0.5°C in an incubator in the dark for stationary growth for 30 d [54]. In total, five repeats of Mb7 and MbK were used, and three of MbK-*swc4*-mutant. At the end of incubation, liquid from each flask was separately transferred through miracloth into a sterile container, separated into two 50-mL tubes and kept at −20°C for further analysis. A single tube from each repeat was subjected to LC – MS analysis, while the other served as a pool for insect-injection bioassays.

## LC – MS analysis

A 1-mL aliquot of each biological replicate was used for spectrometric analysis, using an Agilent HP 1260 Infinity Series liquid chromatography instrument coupled to a Q-TOF mass spectrometer with a Dual ESI source of ionization, and equipped with a DAD system (Agilent Technologies, USA) following a previously described setup [55]. An Adamas C-18 column (4.6 × 50 mm, 3.5 µm, SepaChrom, Italy) was used for the chromatography.

## Statistical analysis of LC – MS data

Statistical analysis of the metabolomics data was carried out by Mass Profile Professional software, version 13.1.1 (Agilent Technologies). Raw data of biological and technical replicates were grouped by isolate and mutant (Mb7, MbK and MbK-*swc4*-mutant) and the groups were subjected to one-way ANOVA and FC ≥ 2.0 analyses. The results were then subjected to PCA and depicted as hierarchical clusters. Relevant statistically significant compounds were identified using an in-house fungal metabolite database, and by comparison with data available in the literature.

## Insect rearing and bioassay

*Galleria mellonella* larvae were reared in sterilized glass jars, fed every 2–3 d and occasionally split to avoid overcrowding and disease development. The jars were maintained in a 25 ± 2°C chamber, with a 12:12 h light regime and an air drier to maintain low humidity.

### Mortality bioassay in *G. mellonella*

For fungal virulence bioassay of MbK and Mb7 compared to the mutants, conidia were collected from 14-d-old SDA culture plates and mixed in sterile 0.01% Triton X-100 to obtain a uniform spore suspensions. The suspensions concentration were estimated by haemocytometer. Suspensions were then diluted to obtain 1 × 10^7^ conidia/mL for inoculum. Experiments were conducted in 90-mm petri dishes, for inoculation Whatman filter paper wetted with 1 mL of spore suspension or 0.01% Triton X-100 as a negative control was placed within the plates. Five to eight *G. mellonella* 5^th^-stage larvae were placed in each petri dish and left to acquire conidia from the inoculated filter paper. Each treatment was conducted in five experimental plates. All plates were placed in a box with pre-wetted paper on the bottom, sealed with a plastic bag and maintained in a 25°C incubator. Mortality was assessed daily (larvae were detached from their silk and returned to the plate). Cadavers were surface-sterilized with bleach and 70% ethanol, washed with distilled water and kept for conidiogenesis in separate petri dishes in a 28°C incubator. The entire set of experiments was conducted in five independent repeats. For statistical analysis, arcsine-transformed values of mortality were subjected to standard least squares analysis by restricted maximum likelihood (REML) method followed by Tukey HSD post hoc analysis for multiple comparisons among means using JMP Pro software version 16.0.0.

### Injection of cultural filtrates into *G. mellonella*

The pooled metabolite solutions were used for injection bioassays in 5^th^-stage *G. mellonella* larvae to assess the toxicity of each fungal filtrate. Each larvae was inoculated by injection of 10 µL suspension into the pre-last proleg and placed on a moistened Whatman filter paper in a 90-mm petri dish. Injected larvae were monitored for the first 10 min to remove paralysed individuals, which were replaced with new treated larvae. Each treatment was conducted in three technical repeats, each containing 10 injected larvae. As a negative control, larvae were injected with 10 µL sterile SDB. A mock treatment contained three repeats of 10 un-injected larvae. The plates were placed in a box, sealed in a plastic bag and incubated in a 25°C incubator. The entire set of bioassays was conducted three times. Mortality measurement and statistical analysis were performed as described above.

## Results

### Isolates MbK and Mb7 demonstrate low genetic polymorphism

#### Resequencing analysis

**Figure 1.**
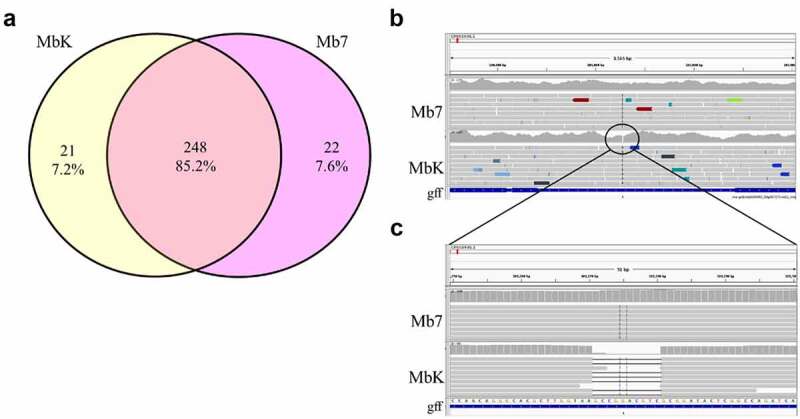
Genetic polymorphism between *Metarhizium brunneum* isolate, Mb7 and MbK. (a) Venn diagram comparing identified mutations in isolates MbK and Mb7 based on reference genome *Metarhizium brunneum* 4556 (Asm1342620v1), conducted by Venny 2.1 tool [56]. (b) Representative snapshot from IVG software showing deletion in MbK genome compared with reference genome and Mb7 isolate. Location in reference genome CP058935.1 nt 101,270. gff: represents gene annotations, upstream to XP_014539572.1 (*MEP1*) and downstream to XP_014539573.1 (*PR1C*) (c) Enlargement of IGV snapshot of the deletion in MbK isolate compared with reference genome and Mb7 isolate.

The obtained data enabled to compare MbK and Mb7 genomes and to seek for nonsynonymous mutations which might explain phenotypic differences observed previously [7]. In the analysis, a small number of single-nucleotide polymorphisms (SNPs) and base insertions/deletions (INDELs) were identified that distinguished between the isolates and the reference genome (*Metarhizium brunneum* 4556; ASM1342620v1). In total, 269 and 270 mutations were found in Mb7 and MbK compared with the reference, respectively. Among these mutations, 248 were present in both isolates including six frameshift variants, four missense variants and one synonymous variant (Figure 1(a); S1 file). The 43 variations distinguishing between the isolates were upstream or downstream of predicted genes (length of 5Kb). None of the identified variations, were located within coding sequence (CDS), and 11 mutations (5 in Mb7 and 6 in MbK) were found to be ±1Kb of predicted genes (Table 1; Figure 1(b)).

**Table 1.** SNPs and INDELs located near predicted genes distinguishing between *M. brunneum* isolates MbK and Mb7.

Location Chromosome (position)	Nearby gene (up- and downstream from mutation) Accession no. of predicted protein in *M. brunneum*	Distance (bp)*	Reference genome**	Mb7	MbK
CP058932.1 (5,610,772)	QLI66165.1 (hypothetical) XP_014543791.1 (stress response protein nst1)	+1083	C	CT	C
	QLI64625.1 (lipase A) XP_014548845.1 (secretory lipase)	+943			
CP058932.1 (9,495,955)	QLI63697.1 (hypothetical) XP_014541215.1 (YhhN-like protein)	+4,272	C	CTT	C
	QLI65247.1 (hypothetical) XP_014541214.1 (oligopeptide transporter)	−50			
CP058933.1 (7,300,466)	QLI67709.1 (grayanic acid biosynthesis cluster O-methyltransferase) XP_014544571.1 (O-methyltransferase)	−119	G	G	A
	QLI66638.1 (serine/threonine-protein kinase gad8) XP_014539489.1 (protein kinase-like protein)	−1765			
CP058934.1 (89,044)	QLI69718.1 (pyranose dehydrogenase) XM_014684893.1 (glucose-methanol-choline oxidoreductase)	+1083	GCCTACATTC	GCCTACATTC	G
	QLI69015.1 (monodictyphenone cluster transcription factor) XP_014540380.1 (aflatoxin biosynthesis regulatory protein)	+768			
CP058934.1 (1,140,468)	QLI70014.1 (alpha-ketoglutarate-dependent taurine dioxygenase) XP_014544201.1 (taurine catabolism dioxygenase tauD/TfdA)	−821	G	G	C
	QLI69251.1 (nonribosomal peptide synthetase fmpE) XP_014544202.1 (pyridoxal phosphate-dependent transferase)	+280			
CP058934.1 (3,332,604)	QLI70028.1 (hypothetical) XP_014542631.1 (FluG domain-containing protein)	+371	C	T	C
CP058934.1 (3,332,605)			A	AATT	A
	QLI69264.1 (hypothetical)	−246			
CP058934.1 (3,332,610)			A	AAACTTAAAAAATATTAAATCATTATATTCTATCTC	A
CP058935.1 (101,270)	QLI71021.1 (hypothetical) XP_014539572.1 (*MEP1*)	−861	AGCCGGACGTC	AGCCGGACGTC	A
	QLI71289.1 (minor extracellular protease vpr) XP_014539573.1 (*PR1C*)	+1150			
CP058936.1 (4,024,430)	QLI71985.1 (hypothetical) XP_014541896.1 (ankyrin repeat protein)	−1023	T	T	TGGGGGGGGGGGGGGG
	QLI72624.1 (hypothetical) XP_014541898.1 (protein kinase-like domain protein)	+1427			
CP058937.1 (280,063)	QLI73557.1 (hypothetical) XP_014540789.1 (hypothetical)	+522	C	C	T
	QLI73412.1 (hypothetical) XP_014540790.1 (hypothetical)	−408			

*Location of mutation in base pair upstream (-) or downstream (+) of gene.

**Reference genome: *Metarhizium brunneum* 4556 (ASM1342620v1).

### *de novo* analysis

**Figure 2.**
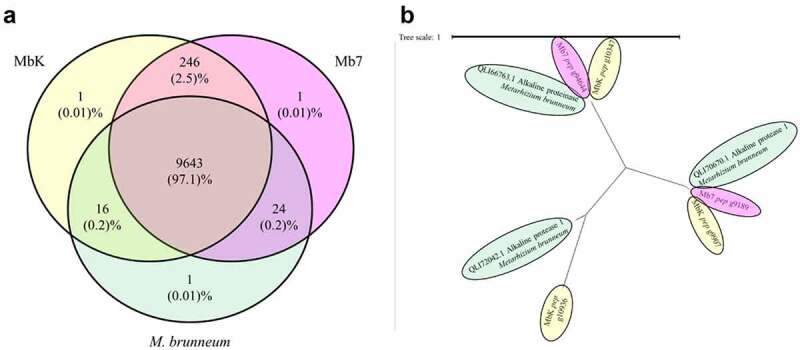
*de novo*analysis of Mb7 and MbK genomes (a) Venn diagram representing the comparison of orthogroups found in Mb7, MbK and the reference genome *Metarhizium brunneum* 4556 (Asm1342620v1), conducted by Venny 2.1 tool [56]. Orthologous proteins were identified with OrthoFinder. (b) Gene tree (using OrthoFinder program) of representative orthogroup (OG0000096; annotation - Alkaline proteinase) for differential gene count between MbK, Mb7 and the reference genome; Scaled by branch length.

Further, we conducted *de novo* analysis in order to identify structural differences that might be missed in the variant calling results. The clean reads were assembled into 348 and 284 scaffolds, for the genomes of Mb7 and MbK isolates, respectively. These were used for gene prediction which revealed minor differences between the isolates, 10,532 genes were identified in Mb7 genome and 10,555 in MbK genome. Comparison of orthogroups between the reference genome and the two isolates, resulted in possible duplications or deletions (S2 file). In the analysis, we detected 56 orthogroups with different copies of genes between the isolates, out of these 40 orthogroups were present only in one isolate: 16 orthogroups in MbK and 24 in Mb7 (Figure 2(a)). Blast2GO analysis on the genes within the unique orthogroups, revealed high similarity in function. Primary metabolic processes demonstrated similar percentage in both isolates (19% and 20% in Mb7 and MbK, respectively), while regulation of primary metabolic processes was identified only within MbK unique orthogroup (S1 Fig). Genes encoding Trypsin were found in two orthogroups, OG0000446 – containing only 1 gene in Mb7 isolate, and OG0000103 – containing two genes in Mb7 and 3 genes in MbK isolate (S2 File). Interestingly, the orthogroup related to protease enzymes (OG0000096) included three genes in the reference and in MbK isolate, while only two genes were identified in Mb7 genome (Figure 2(b); S2 File). Similar protease activity was not found in other orthogroups.

## Adenine methylation in *M. brunneum* isolates

Methylated-adenine sequence libraries were constructed from MbK and Mb7 genomic (g) DNA. Based on the obtained size variation in the colony PCR performed on the obtained bacterial colonies (S2 Fig), 102 colonies were selected for further sequencing. Of these 102 sequences, 38 were collected from an infected aphid library, which mainly provided bacterial sequences from aphids’ symbionts. Other libraries provided all *M. brunneum*-related sequences and all, except one, were sequences within genes (a partial list of obtained genes is presented in S2 Table).

## Differential methylation and expression of *swr1 complex protein 4* (*swc4*) between fungal isolates and growth stages

**Figure 3.**
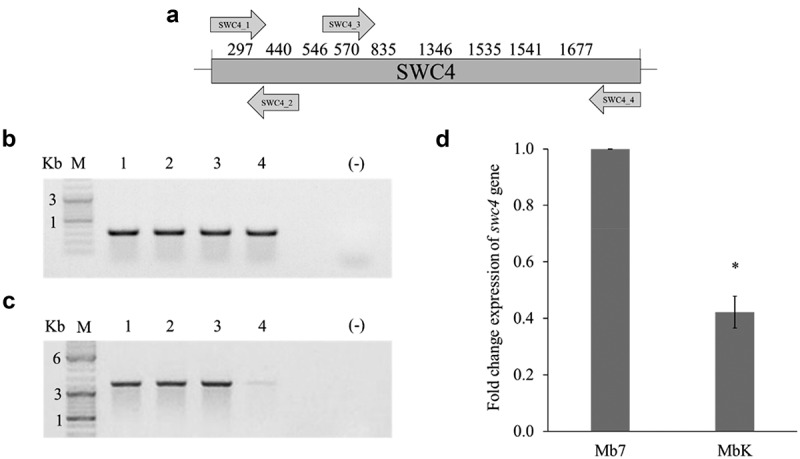
Validation of adenine methylation in the *swc4* gene. (a) Schematic representation of the entire *swc4* gene (nt 1–1971). Numbers above the scheme represent locations of GATC sites in the sequence. Arrows represent primers by orientation and location within the sequence. (b) Agarose (1%) gel demonstrating amplification using SWC4_1 and SWC4_2 primers, giving an amplicon of 324 bp. (c) Agarose (1%) gel demonstrating amplification using SWC4_3 and SWC4_4 primers, giving an amplicon of 1248 bp. Samples: M − 1 kb DNA ladder; 1 – undigested Mb7 gDNA; 2 – undigested MbK gDNA; 3 – *DpnI*-digested Mb7 gDNA; 4 – *DpnI*-digested MbK gDNA. (d) Differential expression of *swc4* between *M. brunneum* isolates Mb7 and MbK tested by qRT-PCR using *translation elongation factor 1 alpha (tef)* as a reference. Asterisk represents statistical significance by *t*-test analysis (*p* <0.05).

Further analysis was carried out on the *swc4* gene, which was identified in the saprophytic conidia library of the MbK isolate. Observation of the known sequence of *swc4* revealed nine sites of GATC which might be methylated on adenine and detectable by *DpnI* digestion (Figure 3(a)). Amplification using SWC4_1 and SWC4_2 primers, generated a 324-bp amplicon which was demonstrated repeatedly in both digested and undigested gDNA from Mb7 and MbK isolates, meaning no cleavage by *DpnI* and thus no adenine methylation on the GATC site (Figure 3(b)). In the amplification using SWC4_3 and SWC4_4 primers, generating a 1248-bp amplicon, differential amplification was observed. Whereas in the Mb7 isolate, similar amplification was detect in digested and undigested gDNA samples, in all three repeats conducted on the MbK isolate, lower amplification of this region was detected in the *DpnI-*digested gDNA (Figure 3(c)). Digestion of the same gDNA with *DpnII*, which detects unmethylated GATC sites, revealed no amplification in any of the repeats (data not shown). This might have resulted from one GATC site not being methylated. To examine the expression of *swc4* and to link the detected methylation to gene regulation, we conducted quantitative (q) RT-PCR on MbK and Mb7 RNA extracted from conidia. MbK demonstrated expression of *swc4*, however with 60% reduction compared to Mb7 (*t*-test: DF = 1, t Ratio = 10.25; *p* < 0.0001, Figure 3(d)).

## Cas9-mediated transformation and mutant selection

**Figure 4.**
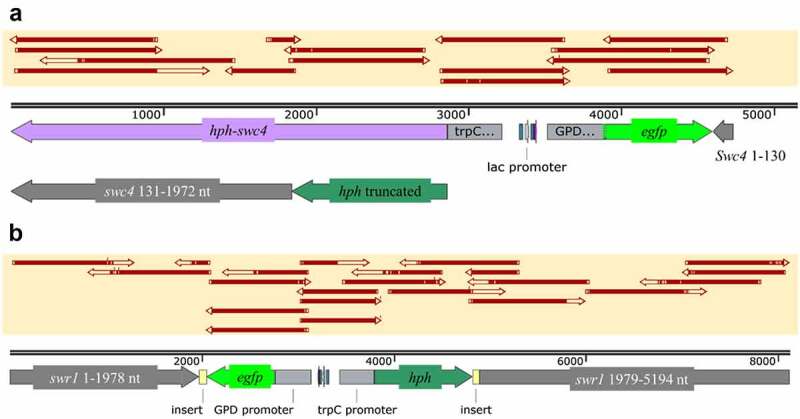
Schematic representation of the mutants generated in this study. (a) *swr1 complex protein 4* (*swc4*) with integration of *hph-gfp* starting from nt position 132 of *swc4*, *hph* stop codon deleted, and *swc4* remaining in-frame until the native stop codon at nt position 1972 of the gene. (b) *swr1* gene with integration of *hph-gfp* starting from nt position 1978, *hph* stop codon present at its original location and before nt position 1979 of *swr1*. Top yellow-shaded boxes in both panels represent PCR sequence alignments.

Mutagenesis of *swc4* was performed with guided RNA located at nt position 132 (S1 Table, SWC4_sg132) and repeated four independent times. Out of over 100 mutants collected, 26 were further transferred to new Sabouraud dextrose agar (SDA) plates containing the antibiotic hygromycin. Eight colonies were unable to grow and were discarded. The other 18 colonies were used for gDNA extraction and sequencing of the *swc4* region in the genome. Only one single colony did not demonstrate integration of the construct hygromycin phosphotransferase-green fluorescent protein gene (*hph-gfp*) within *swc4*. All other mutants had a similar integration site (at 129 nt in *swc4*) with similar distribution of insert orientations (10 in the *swc4* orientation and 7 in reverse orientation). Some of the mutants demonstrated GFP emission under a confocal microscope. However, the signal was too low to be useful as a marker (data not shown). Most of the clones had a deletion in the *gfp* gene. In all mutants, the gene that was oriented as *swc4* (either *gfp* or *hph*) had a deleted stop codon. None of the examined mutants were able to cause a frameshift in *swc4* or to add an early stop codon; rather, the reading frame was left intact after the integration site and until the native stop codon of *swc4* (Figure 4(a)). A single repeat was carried out using another guided RNA at nt position 538 which is located in the SANT domain of the protein. None of the examined colonies demonstrated integration of the insert in *swc4*, and this reaction was therefore not repeated (data not shown).

Mutagenesis of the non-essential SWR1 protein, which is part of the same cellular complex [57], was carried out with guided RNA designed for nt position 1979 in *swr1* (S1 Table, SWR1_sg1979). Twenty-one colonies were collected following transformation, of which a single colony was unable to grow on SDA amended with hygromycin. Two colonies were further used for sequencing, and one was confirmed to have the insert integrated at nt position 1979 in *swr1* (Figure 4(b)), whereas in the other colony, no integration into *swr1* was detected. The selected positive colony had no deletions in *gfp, hph* or *swr1*. The stop codon of *hph* was demonstrated in the sequence. Insertion of 63 and 78 bp was detected surrounding the insert downstream of the stop codon of *gfp* and *hph*, respectively. GFP signal was undetectable by confocal microscopy (data not shown).

Analysis of *swc4* expression in the mutant (downstream of the insertion, S1 Table, qMb_SWC4 F + R) compared to isolates Mb7 and Mbk revealed downregulation, although it was not significant. In the *swr1* mutant (downstream of the insertion, S1 Table, qMb_SWR1 F+ R), the gene was significantly downregulated (F[2,4] = 22.86, *p* = 0.0065; Tukey HSD *p* < 0.05) (S3 Fig). In parallel, amplification with primers encompassing the insertion site in both *swc4* and *swr1* (S1 Table, qMb_SWC4 p.c F + R and qMb_SWR1 p.c F + R) occurred only in the isolates Mb7 and MbK (in *swc4* gene: F[2,4] = 1242.32, *p* < 0.0001, *t*-test for all comparisons *p* < 0.0001; in *swr1* gene: F[2,4] = 30.73, *p* = 0.0059, *t*-test comparison: Mb7 vs *swr1-*mutant *p=*0.0029 and MbK vs *swr1*-mutant *p* = 0.0069) (S3 Fig).

## Reduced fungal saprophytic growth in *swc4* and *swr1* mutants

**Figure 5.**
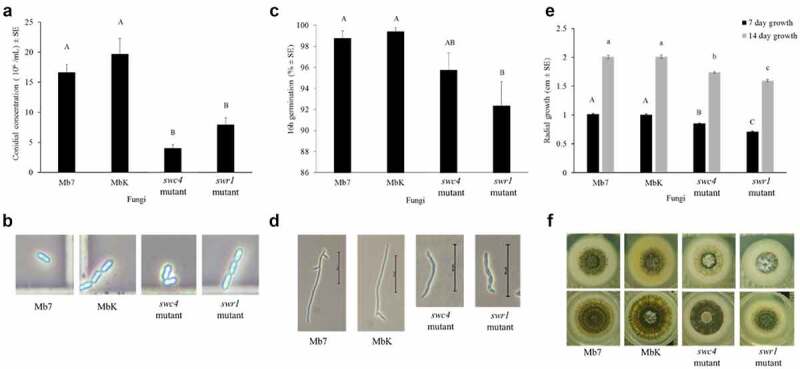
Characterization of fungal morphology and growth. (a) Conidial concentration (1 x 10^6^ conidia/ml ± SE) of the fungal isolates, in a 5-mm agar piece/5 mL Triton X-100 as counted by haemocytometer. (b) Conidia visualized in haemocytometer (enlarged photo of light microscopy, × 400 magnification). (c) Germination rate (% ± SE) of 1 × 10^7^ conidia/ml 16 h after spreading on an SDA plate. (d) Germinated conidia, 16 h after spreading of 1 × 10^7^ conidia/ml on an SDA plate (visualized using light microscopy at × 400 magnification, bar size: 50 µm). (e) Radial growth (cm ± SE) after placing 1 × 10^6^ conidia/ml on the centre of an SDA plate. (f) Radial growth on SDA plates (60 mm) at 7 d (top images) and 14 d (bottom images). Statistical analysis was carried out by Tukey – Kramer test, significant differences are marked with different letters (*p* <0.05).

Morphological characterization of the mutants included visual observation of conidial morphology and sporulation rate, germination and radial growth estimates. During the growth of both fungal mutants, none of the vital processes were arrested; all fungi completed cycles of sporulation, germination and hyphal elongation, but with a significant impact on each stage. Conidial production was significantly reduced by one order of magnitude in the MbK-*swr1*-mutant and MbK-*swc4*-mutant compared to MbK and Mb7, while no difference observed between Mb7 and MbK (F(3,16.08) = 12.05, *p* = 0.0002; Dunnett’s test: *swc4*-mutant *p* = 0.0003 and *swr1*-mutant *p* = 0.003) (Figure 5(a)); however, conidial size and shape did not change (Figure 5(b)). Conidial germination was significantly reduced in both mutants compared to Mb7 and MbK isolates which displayed no difference in germination (F[3,17] = 11.86 *p* = 0.0002; Dunnett’s test: *swc4*-mutant *p=*0.032, *swr1*-mutant *p* = 0.0001) (Figure 5(c)). Hyphal elongation and branching were delayed in both mutants compared to Mb7 and MbK (F(3,26.34) = 520.38, *p<*0.0001; Figure 5(d)) and at both 7 and 14 d of growth, radial colony size was reduced in the *swc4* mutant compared to Mb7 and MbK (Tukey HSD: *p* < 0.0001). The *swr1* mutant demonstrated the most delayed growth, and the difference was significant compared to MbK, Mb7 and the *swc4* mutant (Tukey HSD: *p* < 0.0001) (Figure 5(e,f)).

## Reduced virulence of *swc4* and *swr1* mutants in *Galleria mellonella*

**Figure 6.**
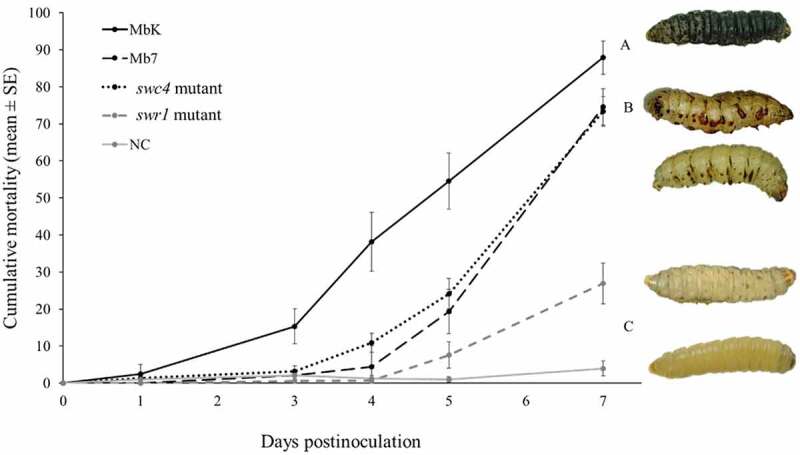
Cumulative mortality (% ± SE) of *G. mellonella* inoculated with spore suspensions of *M. brunneum* isolates MbK, Mb7 and mutants of the genes *swr1* and *swc4* for 7 d postinoculation. Statistical analysis were carried out on arcsine square root-transformed values of mortality proportion using Tukey – Kramer test; significant differences are indicated with different letters (*p* <0.05). On the right: representative larvae infected with (from top to bottom): MbK, Mb7, MbK-*swc4*-mutant, MbK-*swr1*-mutant and non-infected larvae.

To assess the impact of the *swc4* and *swr1* genes on virulence and pathogenicity, bioassays were conducted with the mutants on *G. mellonella* 5^th^-stage larvae. The two mutants were compared to the MbK and the Mb7 isolates. The fungal source material was at 10^7^ spores/mL and germination rates were above 85% in all treatments and repeats. MbK was already able to cause significant mortality of 15% at 3 d postinoculation (DPI; F(4,107.4) = 5.16, *p* = 0.0008). Mb7, MbK-*swc4*-mutant and MbK-*swr1*-mutant caused 19%, 24%, and 8% mortality at 5 DPI, respectively, while the MbK isolate caused over 50% mortality at this time point (F(4, 98.03) = 24, *p* < 0.0001). Finally, MbK caused the highest mortality rate at 7 DPI (90% on average, F(4,88.19) = 70.59, *p* < 0.0001), whereas Mb7 and MbK*-swc4*-mutant caused 75% and 73% mortality, respectively. Mortality of only 27% was achieved with the *swr1* mutant and the difference was not significant compared to the negative control (Fit least square: between isolates (treatment, F(4,739.4) = 75.63 *p* < 0.0001), during time (DPI, F(1,373.9) = 593.69 *p* < 0.0001) and within the treatment*DPI interaction (F(4,736.9) = 67.84, *p* < 0.0001). (Figure 6).

## Metabolomic differences between MbK, Mb7, and MbK-*swc4*-mutant

**Figure 7.**
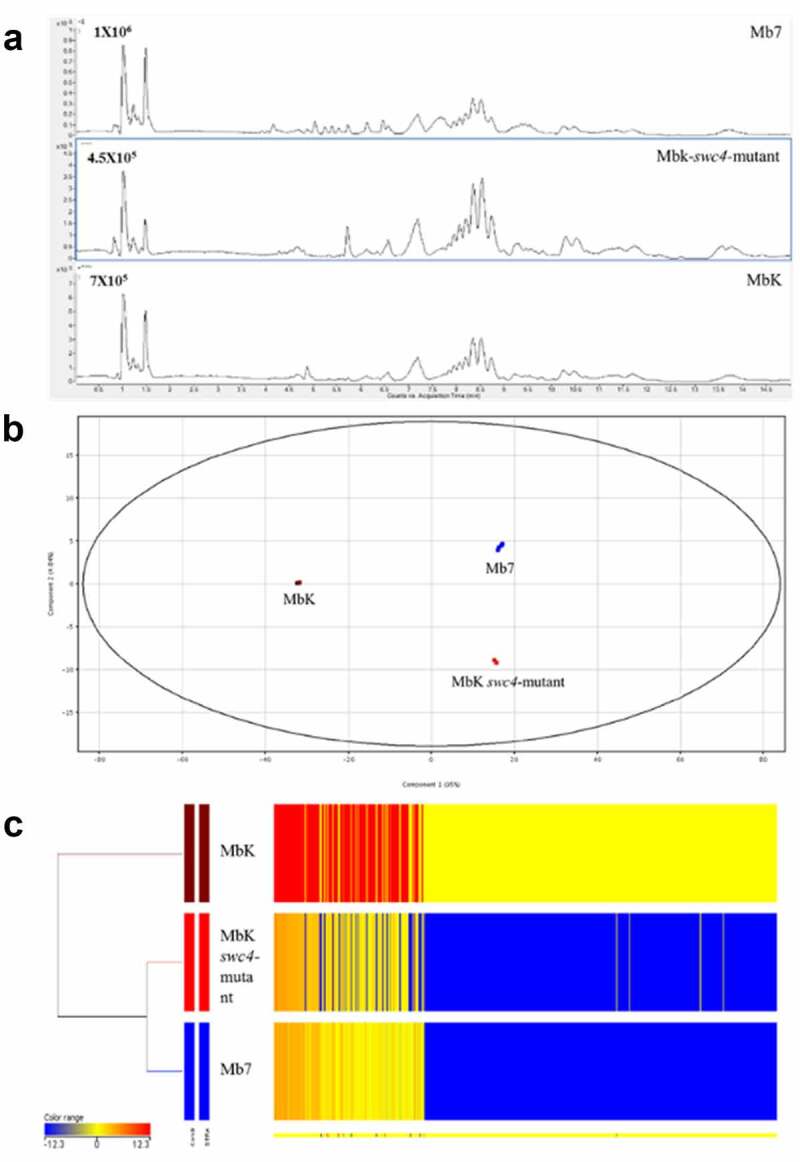
Metabolomic comparison between Mb7, MbK and MbK-*swc4*-mutant cultured in Sabouraud dextrose broth (SDB). (A) Total ion chromatograms of the culture filtrates from Mb7 (top), MbK-*swc4*-mutant (middle) and MbK (bottom). Data were recorded in positive ionization mode. (B) PCA score plot of the LC – MS data acquired in positive mode. Mb7 replicates are depicted in blue; MbK replicates in brown; and MbK-*swc4*-mutant replicates in red. (C) Hierarchal condition tree heatmap of differential metabolic profiles from *M. brunneum* culture filtrates. The abundance of each compound is associated with a colour ranging from blue (less abundant) to red (more abundant).

To further evaluate differences that might explain the variations in virulence, we analysed the metabolomes of the two isolates (Mb7 and MbK) and the *swc4* mutant. Filtrates from Mb7, MbK and MbK-*swc4*-mutant cultures were subjected to LC – MS analysis. Using the resulting data, including total ion chromatograms (Figure 7(a)) and mass spectra, and by comparison with an in-house fungal database and reported data [58], SMs produced by the *Metarhizium* spp. were identified (Table 2). The data included swainsonine, serinocyclin A and B, and a set of destruxins [59,60]. Statistical analysis of the complete SM data from each of the fungal isolates enabled their separation into three groups by principal component analysis (PCA) (Figure 7(b)). In this analysis, 95% of the differences (PC1) contributed to a major separation between MbK and both Mb7 and MbK-*swc4*-mutant. The difference observed between Mb7 and MbK-*swc4*-mutant contributed 5% (PC2, Figure 7(b,c)). In total, the levels of 538 SMs were detected as significantly different between at least two samples (S3 File). Fold-change (FC) analysis between the samples revealed: (a) 503 (93%) metabolites with common levels between Mb7 and Mbk-*swc4*-mutant (−3 < log FC < 3); (b) 521 (97%) SMs that were at higher levels in MbK compared to Mbk-*swc4*-mutant and (c) 506 (94%) SMs that were at higher levels in MbK compared to Mb7 (S3 File). However, among all significantly different metabolites, three were identified by comparison with a fungal database and with the literature (Table 3).

**Table 2.** Identified metabolites of *Metarhizium* spp. obtained by LC – MS analysis.

Compound	RT (min)*	Experimental mass (Da)^¥^	Theoretical mass (Da)^¥^	Molecular formula
Swainsonine	1.033	173.106	173. 10,519,334	C_8_H_15_NO_3_
Serinocyclin A	1.356	672.3094	672. 30,786,887	C_27_H_44_N_8_O_12_
Serinocyclin B	1.477	656.3179	656. 31,295,425	C_27_H_44_N_8_O_11_
Destruxin E-diol	4.348	611.3531	611. 35,302,816	C_29_H_49_N_5_O_9_
Destruxin D	5.537	623.3525	623. 35,302,816	C_30_H_49_N_5_O_9_
Destruxin E	5.605	593.3432	593. 34,246,347	C_29_H_47_N_5_O_8_
Destruxin C2	6.129	595.3524	595. 35,811,354	C_29_H_49_N_5_O_8_
Destruxin A	6.134	577.3454	577. 34,754,886	C_29_H_47_N_5_O_7_
Destruxin B2	6.406	579.3588	579. 36,319,892*	C_29†_H_49_N_5_O_7_

*Retention time.

†Mono-isotopic mass.

**Table 3.** Significant differences in metabolites between filtrates from Mb7, MbK, and MbK-*swc4*-mutant (*swc4*) cultured in SDB.

Compound	log_2_(fold change)				Differential
	(*swc4* vs. Mb7)	(*swc4* vs. MbK)	(Mb7 vs. MbK)		
Swainsonine	0.84	−16.83	−17.68	Mb7, *swc4* < MbK	
Serinocyclin A	17.97	0.39	−17.58	Mb7 < MbK, *swc4*	
Serinocyclin B	17.10	−0.45	−17.56	Mb7 < MbK, *swc4*	

## Pathogenic activity of Mb7, MbK, and MbK-*swc4*-mutant culture filtrates

**Figure 8.**
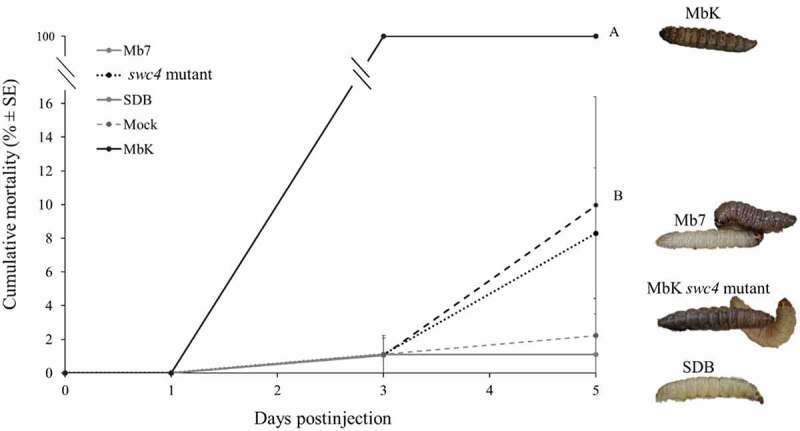
Cumulative mortality (% ± SE) of *G. mellonella* inoculated with filtrate collected from *M. brunneum* isolates MbK, Mb7 and MbK-*swc4*-mutant during 5 d postinjection. Statistical analysis was carried out on arcsine square root-transformed values of mortality proportion using Tukey – Kramer test, and significant differences are indicated by different letters (*p* <0.05). On the right: representative larvae injected with (from top to bottom) MbK, Mb7 and Mbk-*swc4*-mutant filtrate. SDB, Sabouraud dextrose broth.

To evaluate the pathogenic potential of the different filtrates containing SMs from Mb7, MbK, and the mutant of *swc4*, we exposed *G. mellonella* larvae by injecting sterile filtrate directly into their haemocoel without the presence of fungal conidia. A significant difference was observed as early as 3 DPI: larvae injected with MbK filtrate were consistently dead whereas no mortality was observed with the other treatments (F(4,32.58) = 371.88, *p* < 0.0001). Severe melanization occurred on larvae injected with MbK filtrate and was already detectable 1 DPI. At 5 DPI, melanization was also noticeable in some individuals in the other two treatments, but their motility was not affected (Fit least squares: treatment F(4,158.5) = 110.5 *p<*0.0001, DPI F(1,158.6) = 177.25 *p* < 0.0001, treatment*DPI F(4,158.5) = 102.99 *p* < 0.0001) (Figure 8).

## Discussion

We previously showed intra-species variation in disease progression and pathogenicity against aphids between *M. brunneum* isolates MbK and Mb7 [7]. Here, we compared the genetic and epigenetic variation among the isolates to elucidate their role in the varied virulence.

When the genomes of both isolates were compared, we found unexpectedly low genetic variation (SNPs, INDELs) that was evenly dispersed throughout both genomes; we did not detect any highly polymorphic regions [61]. A much higher percentage of polymorphisms between fungal isolates has been observed by others, accompanied by nonsynonymous mutations [61,62]. All of the mutations differentiated MbK from Mb7 were located in intergenic regions. However, mutations located near predicted genes might have a *cis*-regulatory role [63]. An interesting candidate for this type of gene-expression regulation, which might have a role in pathogenicity, is the identified mutation located between two genes, *pr1c* and *mep1*, encoding proteases. These genes play a key role in *Metarhizium*’ s pathogenic lifestyle acting as virulence factors [64,65,66]. Moreover, differential expression was observed in *mep1* gene in response to antifungal agents [6]. Furthermore, *de novo* assembly of Mb7 and MbK genomes revealed high similarity in gene number and functional gene groups. Mainly, absence of specific genes in one of the isolates was a result of grouping similar gene into different orthogroups rather than gene deletion. Interestingly, pathogenicity-related protease orthogroup demonstrated deletion of one gene in the Mb7 isolate. This deletion have to be further studied, possibly by genome editing, in order to elucidate its role in the varied virulence between Mb7 and MbK isolates.

In an attempt to identify an epigenetic variation, we detected 6 mA in the *M. brunneum* genome of both isolates. Specifically, we found the differential presence of 6 mA on *swc4* gene, which was correlated with reduced expression in MbK. We hypothesized that 6 mA plays a regulatory role in *swc4*, which in turn can affect a cascade of events by chromatin remodelling of regions regulating pathogenicity. *Metarhizium* growth as a pathogen requires an arsenal of enzymes and metabolites to conquer the host, especially as the host utilizes rapid epigenetic changes it order to achieve resistance [19]. The fungi should provide a flexible and rapid response which similarly may be achieved through epigenetic modifications [67]. However, in recent publications, the level of 6 mA in true fungi has been shown to be extremely low and its epigenetic regulatory role has been questioned [23,68]. The presence of 6 mA in MbK but not in Mb7 correlated with reduced expression of *swc4* in MbK, as opposed to previous studies [23,69]. This might have been due to other regulatory factors known to work simultaneously [69]. The high distribution of 6 mA in the genomes of prokaryotes, early-diverging fungi, ciliates and algae enables a detailed study of its role in regulating gene expression [23,70,71]. Conversely, in animals, plants and true fungi, further study is required to understand the mode of action and role of 6 mA, despite its low presence in their genomes [68].

Notably, *swc4* might be downregulated to some extent in the MbK isolate and may provide better activity of virulence genes and metabolite secretion, but damaged activity will result in the loss of these abilities as demonstrated in the MbK-*swc4*-mutant. The role of SWC4 subunit, as well as of SWR1-C as a regulator of biological processes in fungi is still largely unknown [32]. Complete knockout of *swc4* was futile, as it repeatedly failed using *Agrobacterium tumefaciens*-mediated transformation by homologous recombination (data not shown). Attempts to mutagenize its locus by *Cas9* resulted in chimeric RNA of *swc4* and *hph* lacking the latter’s stop codon. Putatively, this results in fused protein which might affect SWC4 activity. In contrast, similar mutagenesis of the non-essential *swr1* gene resulted in the chimeric RNA but without the stop codon deletion that repeatedly occurred in *swc4* mutagenesis. These results support the previously reported essential nature of *swc4* [72,73,74,75]. The mutagenesis conducted in this study resulted in significant and extensive damage to the fungus. It may be that the phenotype observed in the *swc4* mutant is due to SWC4 subunit difficulty to bind to chromatin, however that requires further research in the protein level. Interestingly, this type of *Cas9*-mediated transformation can be a useful tool in the study of essential genes which otherwise cannot be manipulated. Moreover, this method provides a bypass for the homologous recombination limit in fungal species possessing Ku proteins for non-homologous end-joining DNA repair [76]. Thus, we present a new venue for studying essential genes.

The results of this study suggest that *swc4* has a significant role in fungal growth, virulence and metabolism, from the immediate growth attenuation in the mutant compared to Mb7 and MbK, to decreased virulence and the significant effect on SM production. It has already been proposed and sporadically proven that SMs are indeed regulated by chromatin remodelling, especially as SM genes are mainly located in clusters which are more efficiently controlled by histone rather than DNA modifications [32,34,77]. The putative disruption of SWC4 protein, and to a larger extent of SWR1-C, can shed light on its regulatory role during the fungus’ life span. As hypothesized by Chen and Ponts (2020), SWC4 mediated histone variant deposition (of H2A.Z) can play a crucial role in biological processes and secondary metabolism in filamentous fungi [32]. Indeed, the SM analysis revealed that the MbK-*swc4*-mutant produces less metabolites but interestingly, this mutation made the MbK isolate, with a single gene disruption, similar to the Mb7 isolate in terms of SM production and virulence. Allegedly, MbK isolate utilizes in parallel both toxin and growth virulence strategies; while Mb7 mainly utilize the growth strategy [7,59]. Further to this hypothesis, *swc4* disruption shifted MbK towards the growth rather than toxin strategy thus pathogenicity was attenuated to some extent but was not lost. Simultaneous elevation of growth and toxicity in a single isolate, may result in a highly virulent EPF.

As subunit SWC4 is part of both SWR and NuA4 complexes [57], whether SM regulation is based on H2A.Z deposition (SWR1-C) or histone acetylation (NuA4 complex), can only be assumed. A much more significant effect on fungal growth, morphology and virulence was observed in the non-lethal *swr1* mutant. Similar morphological effect was already observed in *Candida albicans* and proved to be related to the merge and separation of the two chromatin remodelling complexes, SWR and NuA4, both possessing also the SWC4 subunit [78]. The more severe phenotype in *swr1* mutant could be due to the successful integration of *hph* into the *swr1* gene resulted in RNA sequences encoding probably a truncated protein. We would expect to see, in both mutants, a similar regulatory effect on genes which are regulated by SWR1-C and not by NuA4 (as only SWC4 but not SWR1 is a shared subunit), but this needs to be studied in the future by comparing the transcriptomes of the mutants and MbK. Finally, there remains a large gap in our understanding of the role of 6 mA in gene regulation and its interaction with the SWR-C.

In conclusion, the results of the present study suggest that 6 mA mark in the essential gene *swc4* correlates with downregulation of its expression. SWC4 subunit plays a role in regulation of SMs and SWR1 subunit from the same chromatin remodelling complex is essential for virulence of *M. brunneum*. The main impact of this study lies in promoting the necessary knowledge to manipulate regulators of disease progression in order to increase the efficacy of EPF against agricultural pests. This study opens a new venue of future research possibilities, and provides a useful tool for studying one of the most interesting epigenetic regulation complexes, SWR1-C. A better understanding of SWR1-C can provide new knowledge in other fungi and organisms. This will also expand the possibilities of using genome editing to enhance the performance of *M. brunneum* and other EPF with respect to SMs secretion and pathogenicity against agriculturally important pests.

## Supplementary Material

Supplemental Material

## References

[1] Andreas V. Evolutionary ecology of parasitic fungi and their host insects. Fungal Ecol. 2019;38:12–20.

[2] Roy HE, Steinkraus DC, Eilenberg J, et al. Bizarre interactions and endgames: entomopathogenic fungi and their arthropod hosts. Annu Rev Entomol. 2006;51(1):331–357. 16332215 10.1146/annurev.ento.51.110104.150941

[3] Skinner M, Parker BL, Kim JS. Chapter 10 - Role of entomopathogenic fungi in integrated pest management. In: Abrol D, editor. Integrated pest management [Internet]. San Diego: Academic Press; 2014. p. 169–191. [cited 2021 Nov 25]. Available from: https://www.sciencedirect.com/science/article/pii/B9780123985293000117

[4] van Lenteren JC, Bolckmans K, Köhl J, et al. Biological control using invertebrates and microorganisms: plenty of new opportunities. BioControl. 2018;63(1):39–59.

[5] de Faria MR, Wraight SP. Mycoinsecticides and mycoacaricides: a comprehensive list with worldwide coverage and international classification of formulation types. Biol Control. 2007;43(3):237–256.

[6] Mukherjee K, Vilcinskas A. The entomopathogenic fungus *Metarhizium robertsii* communicates with the insect host *Galleria mellonella* during infection. Virulence. 2018;9(1):402–413. 29166834 10.1080/21505594.2017.1405190 PMC5955202

[7] Reingold V, Kottakota C, Birnbaum N, et al. Intraspecies variation of Metarhizium brunneum against the green peach aphid, Myzus persicae, provides insight into the complexity of disease progression. Pest Manag Sci. 2021;77(5):2557–2567. DOI:10.1002/ps.6294 33486866

[8] Butt TM, Coates CJ, Dubovskiy IM, et al. Entomopathogenic Fungi: New Insights into Host-Pathogen Interactions. Adv Genet. 2016;94:307–364. 27131329 10.1016/bs.adgen.2016.01.006

[9] Vilcinskas A, Matha V, Götz P. Inhibition of phagocytic activity of plasmatocytes isolated from Galleria mellonella by entomogenous fungi and their secondary metabolites. J Insect Physiol. 1997;43(5):475–483. 10.1016/s0022-1910(97)00066-8 12770487

[10] Ment D, Gindin G, Samish M, et al. Comparative response of Metarhizium brunneum to the cuticles of susceptible and resistant hosts. Arch Insect Biochem Physiol. 2020;105(4):e21756. 33140492 10.1002/arch.21756

[11] Ment D, Gindin G, Soroker V, et al. Metarhizium anisopliae conidial responses to lipids from tick cuticle and tick mammalian host surface. J Invertebr Pathol. 2010;103(2):132–139. 20036669 10.1016/j.jip.2009.12.010

[12] Wang J, Lovett B, St Leger RJ. The secretome and chemistry of Metarhizium; a genus of entomopathogenic fungi. Fungal Ecology [Internet]. 2018. [cited 2019 Feb 26]; Available from: http://www.sciencedirect.com/science/article/pii/S175450481830120X

[13] Wang C, St Leger RJ. Developmental and transcriptional responses to host and nonhost cuticles by the specific locust pathogen *Metarhizium anisopliae* var. acridum. Eukaryot Cell. 2005;4(5):937–947. 15879528 10.1128/EC.4.5.937-947.2005 PMC1140090

[14] Ment D, Churchill ACL, Gindin G, et al. Resistant ticks inhibit *Metarhizium* infection prior to haemocoel invasion by reducing fungal viability on the cuticle surface. Environ Microbiol. 2012;14(6):1570–1583. DOI:10.1111/j.1462-2920.2012.02747.x 22507442

[15] Chouvenc T, Su NY, Robert A. Cellular encapsulation in the eastern subterranean termite, *Reticulitermes flavipes* (Isoptera), against infection by the entomopathogenic fungus *Metarhizium anisopliae*. J Invertebr Pathol. 2009;101(3):234–241. 19463828 10.1016/j.jip.2009.05.008

[16] Dubovskiy IM, Whitten MMA, Kryukov VY, et al. More than a colour change: insect melanism, disease resistance and fecundity. Proc R Soc B. 2013;280(1763):20130584. DOI:10.1098/rspb.2013.0584 PMC3774225 23698007

[17] Dombrovsky A, Arthaud L, Ledger TN, et al. Profiling the repertoire of phenotypes influenced by environmental cues that occur during asexual reproduction. Genome Res. 2009;19(11):2052–2063. 19635846 10.1101/gr.091611.109 PMC2775594

[18] Verhoeven KJ, Preite V. Epigenetic variation in asexually reproducing organisms. Evolution. 2014;68(3):644–655. 24274255 10.1111/evo.12320

[19] Mukherjee K, Dubovskiy I, Grizanova E, et al. Epigenetic mechanisms mediate the experimental evolution of resistance against parasitic fungi in the greater wax moth Galleria mellonella. Sci Rep. 2019;9(1):1626. 30733453 10.1038/s41598-018-36829-8 PMC6367475

[20] Gupta AP, Zhu L, Tripathi J, et al. Histone 4 lysine 8 acetylation regulates proliferation and host–pathogen interaction in Plasmodium falciparum. Epigenet Chromatin. 2017;10(1):40. 10.1186/s13072-017-0147-z PMC5568195 28830512

[21] Vilcinskas A. The role of epigenetics in host–parasite coevolution: lessons from the model host insects *Galleria mellonella* and *Tribolium castaneum*. Zoology. 2016;119(4):273–280. 27341739 10.1016/j.zool.2016.05.004

[22] Breiling A, Lyko F. Epigenetic regulatory functions of DNA modifications: 5-methylcytosine and beyond. Epigenet Chromatin. 2015;8(1):24. 10.1186/s13072-015-0016-6 PMC4507326 26195987

[23] Mondo SJ, Dannebaum RO, Kuo RC, et al. Widespread adenine N6-methylation of active genes in fungi. Nat Genet. 2017;49(6):964. DOI:10.1038/ng.3859 28481340

[24] Fu Y, Dominissini D, Rechavi G, et al. Gene expression regulation mediated through reversible m^6^A RNA methylation. Nat Rev Genet. 2014;15(5):293–306. 24662220 10.1038/nrg3724

[25] Low DA, Weyand NJ, Mahan MJ. Roles of DNA adenine methylation in regulating bacterial gene expression and virulence. Infect Immun. 2001;69(12):7197. 11705888 10.1128/IAI.69.12.7197-7204.2001 PMC98802

[26] Pedrini N. Molecular interactions between entomopathogenic fungi (Hypocreales) and their insect host: perspectives from stressful cuticle and hemolymph battlefields and the potential of dual RNA sequencing for future studies. Fungal Biol. 2018;122(6):538–545. 29801798 10.1016/j.funbio.2017.10.003

[27] Woo RM, Park MG, Choi JY, et al. Insecticidal and insect growth regulatory activities of secondary metabolites from entomopathogenic fungi, Lecanicillium attenuatum. J Appl Entomol. 2020;144(7):655–663. DOI:10.1111/jen.12788

[28] Ortiz-Urquiza A, Keyhani NO. Action on the surface: entomopathogenic fungi versus the insect cuticle. Insects. 2013;4(3):357–374. 26462424 10.3390/insects4030357 PMC4553469

[29] Gibson DM, Donzelli BGG, Krasnoff SB, et al. Discovering the secondary metabolite potential encoded within entomopathogenic fungi. Nat Prod Rep. 2014;31(10):1287–1305. 25148015 10.1039/c4np00054d

[30] Pfannenstiel BT, Keller NP. On top of biosynthetic gene clusters: how epigenetic machinery influences secondary metabolism in fungi. Biotechnol Adv. 2019;37(6):107345. 30738111 10.1016/j.biotechadv.2019.02.001 PMC6685777

[31] Zhang L, Fasoyin OE, Molnár I, et al. Secondary metabolites from hypocrealean entomopathogenic fungi: novel bioactive compounds. Nat Prod Rep. 2020;37(9):1181–1206. 32211639 10.1039/c9np00065h PMC7529686

[32] Chen Z, Ponts N. H2A.Z and chromatin remodelling complexes: a focus on fungi. Crit Rev Microbiol. 2020;46(3):321–337. 32594818 10.1080/1040841X.2020.1781784

[33] Cai Q, Tian L, Xie JT, et al. Contributions of a histone deacetylase (SirT2/hst2) to Beauveria bassiana growth, development, and virulence. J Fungi. 2022;8(3):236. 10.3390/jof8030236 PMC8950411 35330238

[34] Atanasoff-Kardjalieff AK, Studt L. Secondary metabolite gene regulation in mycotoxigenic Fusarium species: a focus on chromatin. Toxins (Basel). 2022;14(2):96. 35202124 10.3390/toxins14020096 PMC8880415

[35] Aslam M, Fakher B, Jakada BH, et al. SWR1 chromatin remodeling complex: a key transcriptional regulator in plants. Cells. 2019;8(12):1621. 10.3390/cells8121621 PMC6952815 31842357

[36] Ruhl DD, Jin J, Cai Y, et al. Purification of a human SRCAP complex that remodels chromatin by incorporating the histone variant H2A.Z into nucleosomes. Biochemistry. 2006;45(17):5671–5677. DOI:10.1021/bi060043d 16634648

[37] Baverstock J, Roy HE, Pell JK. Entomopathogenic fungi and insect behaviour: from unsuspecting hosts to targeted vectors. In: Roy H, F Vega, D Chandler, M Goettel, J Pell, E Wajnberg, editors. The ecology of fungal entomopathogens [Internet]. Dordrecht: Springer Netherlands; 2010. p. 89–102. Available from 10.1007/978-90-481-3966-8_7.

[38] Araújo JPM, Hughes DP. Diversity of entomopathogenic fungi: which groups conquered the insect body? Adv Genet. 2016;94:1–39. 27131321 10.1016/bs.adgen.2016.01.001

[39] Li H, Durbin R. Fast and accurate long-read alignment with Burrows–Wheeler transform. Bioinformatics. 2010;26(5):589–595. 20080505 10.1093/bioinformatics/btp698 PMC2828108

[40] Li H, Handsaker B, Wysoker A, et al. The sequence alignment/map format and SAMtools. Bioinformatics. 2009;25(16):2078–2079. DOI:10.1093/bioinformatics/btp352 19505943 PMC2723002

[41] DePristo MA, Banks E, Poplin R, et al. A framework for variation discovery and genotyping using next-generation DNA sequencing data. Nat Genet. 2011;43(5):491–498. DOI:10.1038/ng.806 21478889 PMC3083463

[42] Cingolani P, Platts A, Wang LL, et al. A program for annotating and predicting the effects of single nucleotide polymorphisms, SnpEff. Fly (Austin). 2012;6(2):80–92. DOI:10.4161/fly.19695 22728672 PMC3679285

[43] Prjibelski A, Antipov D, Meleshko D, et al. Using SPAdes De Novo assembler. Curr Protoc Bioinformatics. 2020;70(1):e102. 32559359 10.1002/cpbi.102

[44] Hoff KJ, Stanke M. Predicting genes in single genomes with AUGUSTUS. Curr Protoc Bioinformatics. 2019;65(1):e57. 30466165 10.1002/cpbi.57

[45] Buchfink B, Xie C, Huson DH. Fast and sensitive protein alignment using DIAMOND. Nat Methods. 2015;12(1):59–60. 25402007 10.1038/nmeth.3176

[46] Conesa A, Götz S, García-Gómez JM, et al. Blast2go: a universal tool for annotation, visualization and analysis in functional genomics research. Bioinformatics. 2005;21(18):3674–3676. 16081474 10.1093/bioinformatics/bti610

[47] Emms DM, Kelly S. OrthoFinder: solving fundamental biases in whole genome comparisons dramatically improves orthogroup inference accuracy. Genome Biol. 2015;16(1):157. 26243257 10.1186/s13059-015-0721-2 PMC4531804

[48] Reingold V, Luria N, Robichon A, et al. Adenine methylation may contribute to endosymbiont selection in a clonal aphid population. BMC Genomics. 2014;15(1):999. 25406741 10.1186/1471-2164-15-999 PMC4246565

[49] Fang W, Bidochka MJ. Expression of genes involved in germination, conidiogenesis and pathogenesis in *Metarhizium anisopliae* using quantitative real-time RT-PCR. Mycol Res. 2006;110(10):1165–1171. 17010593 10.1016/j.mycres.2006.04.014

[50] Livak KJ, Schmittgen TD. Analysis of relative gene expression data using real-time quantitative PCR and the 2(-delta delta C(T)) method. Methods. 2001;25(4):402–408. 11846609 10.1006/meth.2001.1262

[51] Davis KA, Sampson JK, Panaccione DG. Genetic reprogramming of the ergot alkaloid pathway of Metarhizium brunneum. Appl Environ Microbiol. 2020;86(19). DOI:10.1128/AEM.01251-20 PMC7499049 32769181

[52] Liu X, Homma A, Sayadi J, et al. Sequence features associated with the cleavage efficiency of CRISPR/Cas9 system. Sci Rep. 2016;6:19675. 26813419 10.1038/srep19675 PMC4728555

[53] Freeman S, Sharon M, Dori-Bachash M, et al. Symbiotic association of three fungal species throughout the life cycle of the ambrosia beetle Euwallacea nr. fornicatus. Symbiosis. 2016;68(1–3):115–128. DOI:10.1007/s13199-015-0356-9

[54] De Filippis A, Nocera FP, Tafuri S, et al. Antimicrobial activity of harzianic acid against Staphylococcus pseudintermedius. Nat Prod Res. 2021;35(23):5440–5445. DOI:10.1080/14786419.2020.1779714 32538678

[55] Staropoli A, Vassetti A, Salvatore MM, et al. Improvement of nutraceutical value of parsley leaves (Petroselinum crispum) upon field applications of beneficial microorganisms. Horticulturae. 2021;7(9):281. DOI:10.3390/horticulturae7090281

[56] Oliveros JV. An interactive tool for comparing lists with Venn’s diagrams [Internet]. 2007 [cited 2022 Mar 13]. Available from: https://bioinfogp.cnb.csic.es/tools/venny/index.html

[57] Pyt L, Lévesque N, Kobor MS. NuA4 and SWR1-C: two chromatin-modifying complexes with overlapping functions and components. Biochem Cell Biol. 2009;87(5):799–815. 19898529 10.1139/O09-062

[58] Molnár I, Gibson DM, Krasnoff SB. Secondary metabolites from entomopathogenic Hypocrealean fungi. Nat Prod Rep. 2010;27(9):1241–1275. 20601982 10.1039/c001459c

[59] Kershaw MJ, Moorhouse ER, Bateman R, et al. The role of destruxins in the pathogenicity of metarhizium anisopliae for three species of insect. J Invertebr Pathol. 1999;74(3):213–223. 10534408 10.1006/jipa.1999.4884

[60] Krasnoff SB, Keresztes I, Gillilan RE, et al. Serinocyclins a and B, cyclic heptapeptides from Metarhizium anisopliae. J Nat Prod. 2007;70(12):1919–1924. DOI:10.1021/np070407i 18044842

[61] Chen C, Lian B, Hu J, et al. Genome comparison of two Magnaporthe oryzae field isolates reveals genome variations and potential virulence effectors. BMC Genomics. 2013;14(1):887. DOI:10.1186/1471-2164-14-887 24341723 PMC3878650

[62] Chen X, Zhang X, Zhu P, et al. A single nucleotide mutation in adenylate cyclase affects vegetative growth, sclerotial formation and virulence of Botrytis cinerea. Int J Mol Sci. 2020;21(8):E2912. DOI:10.3390/ijms21082912 32326350 PMC7215688

[63] Wray GA. The evolutionary significance of cis-regulatory mutations. Nat Rev Genet. 2007;8(3):206–216. 17304246 10.1038/nrg2063

[64] Gao BJ, Mou YN, Tong SM, et al. Subtilisin-Like Pr1 proteases marking the evolution of pathogenicity in a wide-spectrum insect-pathogenic fungus. Virulence. 2020;11(1):365–380. 32253991 10.1080/21505594.2020.1749487 PMC7199741

[65] Bidochka MJ, Melzer MJ. Genetic polymorphisms in three subtilisin-like protease isoforms (Pr1a, Pr1B, and Pr1C) from Metarhizium strains. Can J Microbiol. 2011 Feb 10[cited 2022 Feb 16]; Available from https://cdnsciencepub.com/doi/abs/10.1139/w00-112 11142404

[66] Santi L, Beys da Silva WO, Berger M, et al. Conidial surface proteins of Metarhizium anisopliae: source of activities related with toxic effects, host penetration and pathogenesis. Toxicon. 2010;55(4):874–880. DOI:10.1016/j.toxicon.2009.12.012 20034509

[67] Li CY, Chung YM, Wu YC, et al. Natural products development under epigenetic modulation in fungi. Phytochem Rev. 2020;19(6):1323–1340.

[68] Bochtler M, Fernandes H. DNA adenine methylation in eukaryotes: enzymatic mark or a form of DNA damage? Bioessays. 2021;43(3):2000243. 10.1002/bies.202000243 33244833

[69] Wang Y, Sheng Y, Liu Y et al. A distinct class of eukaryotic MT-A70 methyltransferases maintain symmetric DNA N6-adenine methylation at the ApT dinucleotides as an epigenetic mark associated with transcription. Nucleic Acids Res. 2019;47(22):11771–11789. DOI:10.1093/nar/gkz1053 31722409 PMC7145601

[70] Fu Y, Luo GZ, Chen K, et al. N6-Methyldeoxyadenosine marks active transcription start sites in Chlamydomonas. Cell. 2015;161(4):879–892. DOI:10.1016/j.cell.2015.04.010 25936837 PMC4427561

[71] Luo GZ, Hao Z, Luo L, et al. N6-Methyldeoxyadenosine directs nucleosome positioning in Tetrahymena DNA. Genome Biol. 2018;19(1):200. DOI:10.1186/s13059-018-1573-3 30454035 PMC6245762

[72] Miciałkiewicz A, Chełstowska A. The essential function of Swc4p - a protein shared by two chromatin-modifying complexes of the yeast Saccharomyces cerevisiae - resides within its N-terminal part. Acta Biochim Pol. 2008;55(3):603–612. 18726008

[73] Gómez-Zambrano Á, Crevillén P, Franco-Zorrilla JM, et al. Arabidopsis SWC4 binds DNA and recruits the SWR1 complex to modulate histone H2A.Z deposition at key regulatory genes. Mol Plant. 2018;11(6):815–832. DOI:10.1016/j.molp.2018.03.014 29604400

[74] Salas-Santiago B, Lopes JM. Saccharomyces cerevisiae essential genes with an opi− phenotype. G3 genes|genomes|genetics. 2014;4(4):761–767. DOI:10.1534/g3.113.010140. PMC4059245 24558266

[75] Giaever G, Chu AM, Ni L, et al. Functional profiling of the Saccharomyces cerevisiae genome. Nature. 2002;418(6896):387–391. DOI:10.1038/nature00935 12140549

[76] Xu C, Zhang X, Qian Y et al. A high-throughput gene disruption methodology for the entomopathogenic fungus Metarhizium robertsii. PLoS One. 2014;9(9):e107657. DOI:10.1371/journal.pone.0107657 25222118 PMC4164657

[77] Albright JC, Henke MT, Soukup AA, et al. Large-scale metabolomics reveals a complex response of Aspergillus nidulans to epigenetic perturbation. ACS Chem Biol. 2015;10(6):1535–1541. DOI:10.1021/acschembio.5b00025 25815712 PMC4475433

[78] Wang X, Zhu W, Chang P, et al. Merge and separation of NuA4 and SWR1 complexes control cell fate plasticity in Candida albicans. Cell Discov. 2018;4(1):45. 30109121 10.1038/s41421-018-0043-0 PMC6089883

