# Supplementary figures and images for: The SWC4 subunit of the SWR1 chromatin remodeling complex is involved in varying virulence of Metarhizium brunneum isolates offering role of epigenetic regulation of pathogenicity
Source: Virulence. 2022 Jul 26;13(1):1252–69. doi: 10.1080/21505594.2022.2101210 (PMC9336478; doi:10.1080/21505594.2022.2101210)

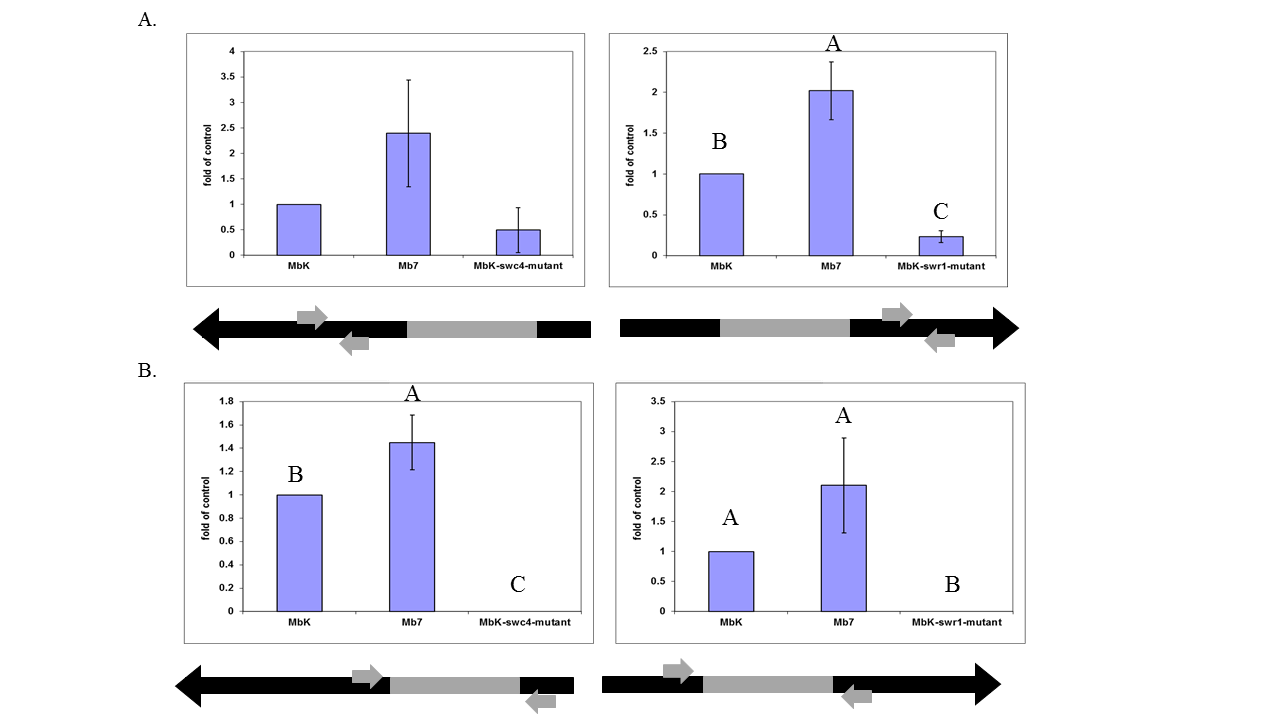

Supplement: Supplemental Material [file KVIR_A_2101210_SM8114.zip › supplementary/F S3.tif]
